# Developing Health Promotion Interventions on Social Networking Sites: Recommendations from The FaceSpace Project

**DOI:** 10.2196/jmir.1875

**Published:** 2012-02-28

**Authors:** Judy Gold, Alisa E Pedrana, Mark A Stoove, Shanton Chang, Steve Howard, Jason Asselin, Olivia Ilic, Colin Batrouney, Margaret E Hellard

**Affiliations:** ^1^Centre for Population HealthBurnet InstituteMelbourneAustralia; ^2^Department of Epidemiology and Preventive MedicineMonash UniversityMelbourneAustralia; ^3^Department of Information SystemsThe University of MelbourneMelbourneAustralia; ^4^Victorian AIDS Council/Gay Men’s Health CentreMelbourneAustralia; ^5^X:MACHINE ProductionsMelbourneAustralia; ^6^The Nossal Institute for Global HealthThe University of MelbourneMelbourneAustralia

**Keywords:** Health promotion, Internet, social networking sites

## Abstract

Online social networking sites offer a novel setting for the delivery of health promotion interventions due to their potential to reach a large population and the possibility for two-way engagement. However, few have attempted to host interventions on these sites, or to use the range of interactive functions available to enhance the delivery of health-related messages. This paper presents lessons learnt from “The FaceSpace Project”, a sexual health promotion intervention using social networking sites targeting two key at-risk groups. Based on our experience, we make recommendations for developing and implementing health promotion interventions on these sites. Elements crucial for developing interventions include establishing a multidisciplinary team, allowing adequate time for obtaining approvals, securing sufficient resources for building and maintaining an online presence, and developing an integrated process and impact evaluation framework. With two-way interaction an important and novel feature of health promotion interventions in this medium, we also present strategies trialled to generate interest and engagement in our intervention. Social networking sites are now an established part of the online environment; our experience in developing and implementing a health promotion intervention using this medium are of direct relevance and utility for all health organizations creating a presence in this new environment.

## Introduction

Over the past 20 years the Internet has dramatically changed how individuals access information and communicate. Global Internet use has grown exponentially, with an estimated 1.8 billion Internet users in 2009, up from 318 million users in 1998 [1]. The Internet is increasingly used for health purposes [2]; one survey reported 83% of American Internet users source health information online [3]. Numerous Internet-based health interventions have been developed, with several reviews concluding that such interventions generally have positive effects for a range of behaviours [4-7].

‘Web 2.0’ is a relatively recent development that refers to a loose collection of web-based technologies and services that allow end users to interact and collaborate as content creators, rather than the one-way information flow on relatively static ‘Web 1.0’ websites [8-10]. The term ‘social media’ is used interchangeably with Web 2.0 to describe sites and applications that allow information sharing and interactive activities among online communities; examples include blogs, wiki’s, content-sharing sites, virtual worlds and social networking sites [10,11]. 

Social networking sites allow individuals to maintain, form and visualize their social networks, and often offer additional functions such as public and private messaging and photo, video and other content sharing [12]. Facebook, Twitter, LinkedIn and MySpace are the most popular social networking sites globally [13], with others largely popular only within certain sub-groups or geographical regions [12]. Growth in usage has been extremely rapid, with Facebook reporting 500 million active users [14], up from 200 million in April 2009 [15]. 

Commercial organizations have been quick to capitalize on the utility of using Web 2.0 to attract, retain and engage end users [10], while health organizations have lagged behind [10,16,17]. Very little has been published about how social networking sites might be exploited for health promotion interventions. A recent review of the use of social media for social marketing identified just four examples, none of which used the most common social networking sites listed earlier [18]. Some health organizations have begun extending their presence into social networking sites [19-22]; however, this has often been used as an additional form of marketing to promote services rather than for intervention delivery. Other work has focused on the public display of risky behaviour (e.g. alcohol use) on these sites [23,24]. However, there are few published examples of organizations actually *delivering* health promotion interventions through social networking sites.

The lack of published examples describing intervention delivery using social networking sites makes it very difficult for others to realistically consider if and how they might approach developing interventions in these spaces. Moreover, the lack of evidence for evaluating such interventions makes it difficult to determine if health promotion interventions using social networking sites are effective.

During 2009 and 2010, we implemented a novel health promotion intervention using social networking sites: “The FaceSpace Project”. The aim of this paper is to use our experience to provide recommendations for developing health promotion interventions on social networking sites.

### The FaceSpace Project

The FaceSpace Project trialled the delivery of sexual health promotion via social networking sites to two key at-risk groups: young people aged 16-29 years, and men who have sex with men (MSM). The project concept was to use fictional characters to post content (primarily videos) and to interact on various social networking sites, with sexual health promotion messages embedded within some postings and interactions. The project was a collaboration between public health researchers, experts in user interaction with information technologies, a creative productions company, and a community organization.

**Figure 1:**
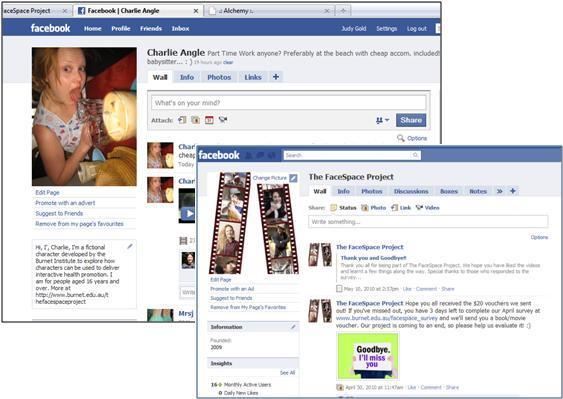
Screenshots from the arm of The FaceSpace Project targeting young people.

The young people’s arm was developed and implemented first. Two young male and two young female characters and character narratives were developed in workshops with young people, actors, and project staff, and character narratives developed. Each character had a Facebook page (www.facebook.com/thefacespaceproject), and a presence on one other social networking site (Twitter, Flickr, YouTube) (www.youtube.com/thefacespaceproject) (Figure 1). (Note that the pages for the young people’s arm are no longer actively maintained.) The overall project also had a Facebook page and a YouTube channel. From November 2009 until April 2010, each character posted regular updates and periodic videos on their sites, including interactions on each other’s sites. Project evaluation included site usage and interaction statistics, questionnaires and focus group discussions.

**Figure 2:**
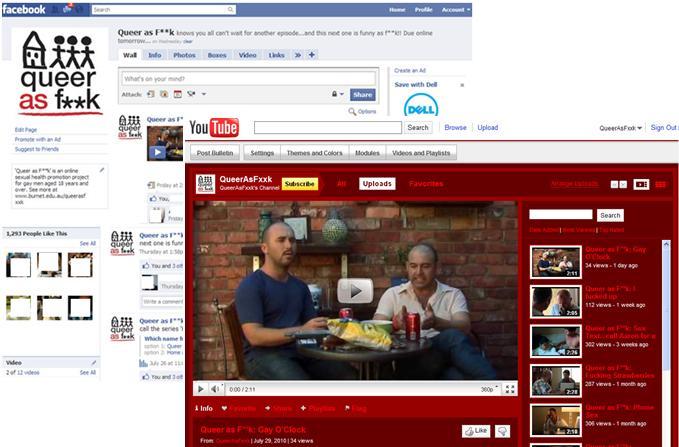
Screenshots from the arm of The FaceSpace Project (Queer As F**k) targeting men who have sex with men.

The learnings from the young people’s arm of the project informed the development of the MSM arm. This arm was separately branded ‘Queer As F**k’ and launched online in April 2010. In this arm, four male characters (all MSM) were developed; however, the emphasis of the development phase was on the series narrative (rather than character-focused), and all the characters interacted together on one Facebook page (www.facebook.com/QAFxxk), supported by one YouTube channel (www.youtube.com/queerasfxxk) (Figure 2). Unlike the youth arm where the videos were styled predominantly as personal blogs, videos in the MSM arm were episodic in nature with a cohesive narrative and sexual health themes embedded in most episodes. Similar evaluation methods were used to the youth arm, with the addition of a diary-scrapbook.

### Key Outcomes From The FaceSpace Project

**Figure 3:**
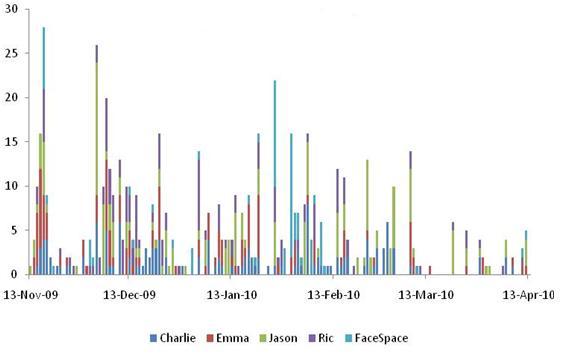
Daily number of interactions (‘likes’, wall posts and comments) on the Facebook pages of the arm of The FaceSpace Project targeting young people.

At the conclusion of the young people’s arm of the project, the 5 Facebook pages had a total of 900 fans. The 31 project videos had 5300 total views on YouTube, with views of individual videos ranging from 12 to 3188 views. Interaction on the Facebook pages varied over time (Figure 3), with peaks generally corresponding to posting of project videos.

**Figure 4:**
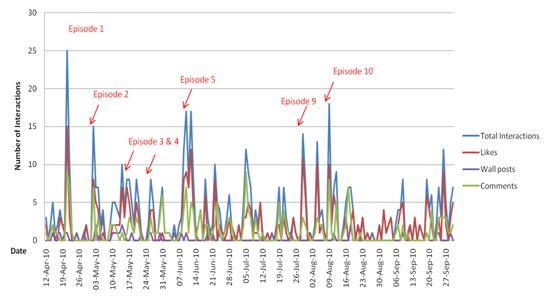
Daily number of interactions (‘likes’, wall posts and comments) on the Facebook page of the arm of The FaceSpace Project targeting men who have sex with men (Queer As F**k).

At the conclusion of the arm of the project targeting gay men (‘Queer as F**k’), the Facebook page had 1332 fans. The 10 video episodes of the project had 7886 views, with views of individual episodes ranging from 256 to 1814 views. As with the youth arm of the project, interaction on the Facebook page varied over time, with peaks in interactions generally corresponding to when new video episodes were posted (Figure 4).

Since the conclusion of The FaceSpace Project, the arm targeting gay men (‘Queer as F**k’) has been taken up by the project’s key community partner, the Victorian AIDS Council/Gay Men’s Health Service. Subsequent seasons of Queer as F**k now form an integral part of their social marketing campaigns. Findings from The FaceSpace Project have guided modifications to these subsequent seasons by using more implicit sexual health messages embedded within dramatic threads, and by creating an online environment for organic user-led, rather than expert-led, dialogue.

### Key Recommendations from The FaceSpace Project

Key recommendations from the FaceSpace Project are shown in Table 1.

**Table 1:** Recommendations from The FaceSpace Project.

	Recommendation
1	Create and nurture a multidisciplinary team with all the skills required—just because you can drive a car and change the oil doesn’t make you a mechanic
2	Anticipate delays getting approval (ethical, legal, organizational)—it’s a new medium and sometimes the waters haven’t been tested
3	Resource, resource, resource—you will need time, money, human and brain power to develop and maintain sites (without forgetting the rest)
4	Generate interest (buzz) and do it early—just because you’ve built it, doesn’t mean they’ll come
5	Keep your audience engaged—don’t fall off the newsfeed!
6	Go viral—if you find the formula, you’re a millionaire
7	Define success and how you will measure it

#### Recommendation 1. Create and Nurture a Multidisciplinary Team With All the Skills Required—Just Because You Can Drive a Car and Change the Oil Doesn’t Make You a Mechanic.

Unlike standard health promotion interventions where many organizations have the in-house expertise required for implementation, interventions on social networking sites require additional expertise in social media and knowledge of how end users interact and engage in online environments. Familiarity with social networking sites from personal experience is insufficient to build and maintain an organization presence or to design a health promotion intervention in these spaces. Teams require a broad range of skills and knowledge, including an adequate understanding of potential sites and their functionality as well as an understanding of social marketing.

We formed a multidisciplinary project team that involved public health researchers (Burnet Institute), experts in how end users interact with technology (Department of Information Systems, University of Melbourne), a creative productions company experienced in online performances (X:MACHINE) and a community organization experienced in sexual health promotion (Victorian AIDS Council/Gay Men’s Health Centre). An advisory group comprised of experts in various fields related to the project was also convened to provide ongoing advice.

Although our multidisciplinary project team was successfully established, such collaborations bring difficulties of their own, including ensuring timely and adequate communications, clear delineation of roles and responsibilities, and interdisciplinary tensions including different philosophies underpinning approaches to design and implementation (eg, user-led vs creative-led design). This was the first time this team had worked together, and we had not anticipated the resources (time, financial) required to build and maintain this collaboration. Such resourcing is vital to ensure a healthy and vibrant collaboration to support the development of effective interventions.

#### Recommendation 2. Anticipate Delays Getting Approval (Ethical, Legal, Organizational)—It’s a New Medium and Sometimes the Waters Haven’t Been Tested.

The use of social networking sites for health promotion interventions can raise ethical, legal and organizational concerns. In addition, individuals and boards who are responsible for approving interventions may not be familiar with social networking sites or how they are used by individuals [25]. Potential concerns include privacy, consent, intervention access, duty of care, organizational reputation, data collection and management, and reduced control over message delivery compared to other settings.

In our case, while legal approval was relatively straightforward, we had some challenges negotiating intellectual property ownership between the collaborating organizations. In addition, we underwent a lengthy review process before being granted approval by our ethical review board. One positive outcome of this review included development of a clearer and more detailed protocol for responding to ‘inappropriate’ posts on our pages (see Multimedia Appendix 1). However, we were required to significantly modify the delivery of our intervention in several ways. For example, the board required prominent disclaimers on the page and regular reminders to fans that reinforced that our characters were fictional, and warnings to not post information that individuals ‘may regret later’. We believe these requirements may have negatively impacted on our credibility on social networking sites and thus reduced end users’ willingness to participate and engage with our intervention.

Social networking sites are a new and challenging environment for many organizations. This should be anticipated in project timelines, as applying and obtaining ethical, legal and organizational approval can be time-consuming and difficult. Content areas considered socially ‘sensitive’ (such as ours) or related to illicit behaviour (eg, drug use) may attract additional scrutiny, given the public nature of social networking sites. Including a “Social Networking 101” education component for approval bodies during the development period may be a useful strategy to minimise delays in obtaining approval.

#### Recommendation 3. Resource, Resource, Resource—You Will Need Time, Money, Human and Brain Power to Develop and Maintain Sites (Without Forgetting the Rest).

One of the advantages of delivering health interventions online is they can reach a large number of people relatively cheaply, and at a reduced cost compared to other approaches [2,26]. However, although hosting pages on social networking sites is free, the time spent creating, developing and maintaining them isn’t. The time to upload posts can be substantial when multiple sites need to be updated, and posts monitored and responded to. Sourcing and developing the content of posts also requires resources; even if sites are largely reliant on existing content, this must be sourced and reviewed for accuracy and appeal. We used an ‘edutainment’ (education and entertainment) approach to maximize appeal to our target audience, which required substantial investment to develop.

As our project involved a novel approach, we were unsure at the outset of the resources required. As the project evolved, we realized we had substantially under-estimated the time and effort required to develop the sites initially, and to maintain them for the duration of the project. Given the amount of information on social networking sites, and the speed at which information changes (on Facebook alone 30 billion pieces of content are shared each month [14]), we potentially needed to be posting content several times a day, rather than every few days. We found that the resources required to maintain sites detracted from attending to other key tasks, such as exploring alternative approaches to engage end users, maintaining our collaboration, and project evaluation. Upon reflection, it would have been ideal to have had the capacity to employ an individual with the time and interest to maintain the pages (eg, an avid social media user), rather than using a combination of creative professionals and researchers, whose primary project roles were not online maintenance.

#### Recommendation 4. Generate Interest (buzz) and Do It Early—Just Because You’ve Built It, Doesn’t Mean They’ll Come.

One of the greatest challenges for health promotion interventions using social networking sites is being noticed amongst the huge amount of content online. Unlike traditional advertising, being visually appealing is not sufficient to attract attention. It helps to have an established base of end users when the site is launched; feedback from our initial IT laboratory testing with end users indicated sites need to look active to attract interest from others. We attempted to do this by ‘soft launching’ our pages via word-of-mouth through personal and professional networks. However, this approach risked having an initial fan base different to the target demographic, which may limit the appeal of the intervention to the intended audience.

Promotion of the intervention is also critical; while we utilized ‘traditional’ methods of promotion such as print and broadcast media coverage and advertising, by far the most successful was using Facebook advertisements (although ours had an incentive attached) and uploading and tagging photos of end users at public events. Others have also noted the success of using online advertisements and photo tagging to attract end users [22,27]. Resources for promotion are most effectively spent in online strategies that allow end users to immediately ‘click through’ to sites, rather than a two-step process of viewing the advertisement and finding the site online. Having a defined offline community to reach (as we did for the MSM arm of the project) also assists with targeting promotion.

#### Recommendation 5. Keep Your Audience Engaged—Don’t Fall Off the Newsfeed!

Users frequently connect with pages and groups, and download applications, never to take notice of them again. The amount of content available is overwhelming; Facebook alone has 900 million pages, groups, events and community pages [14]. This presents a difficulty for the delivery of health promotion online, especially when sustained engagement over time is required to deliver the intervention.

We were conscious at the outset that we did not want to deliver a Web 1.0 intervention using a Web 2.0 site. We aimed to truly interact and engage with our target group, not just broadcast information. The challenge was to maintain interest and engagement over a four-month intervention period with sufficient audience reach. We attempted to do this by using different delivery mechanisms such as posting regular updates (both text and videos), posing questions and encouraging comments on posts, and launching quizzes and polls, with varying success. However, it was clear from site usage data that interest and interaction on our pages declined considerably over time in both project arms (Figure 3 and Figure 4). In addition, the use of multiple delivery mechanisms may have ‘fragmented’ our key health messages; even if an individual had been exposed to one delivery mechanism, they may not have received the full message if they did not view other content on the site.

The loss of participants over time within an online intervention – the ‘Law of Attrition’ – is well known, and is often simply due to loss of interest of participants [10,28]. In retrospect, we may have been able to increase (or simply maintain) engagement by delivering the intervention over a shorter time frame, focusing on a single core message, ensuring all posts could act as ‘stand alone’ messages and creating more opportunities for end users to generate and manipulate content themselves. Further investigations are needed to establish the optimum methods to engage users of social networking sites in health promotion interventions, and to retain them over time.

#### Recommendation 6. Go Viral—If You Find the Formula, You’re a Millionaire.

Ultimately, the greatest success one can have on a social networking site is “going viral” —where enough people are sufficiently interested in a post to share it with their friends, who then share it with their friends and so on, resulting in an exponential growth of connections. This spread of information has been termed ‘Internet meme’ [29]; the most common examples are when videos go viral and attract millions of views (eg, “Dancing Matt”, “Obama Girl”, “Diet Coke + Mentos” [30]). Even some videos containing health-related content have managed to achieve this; for example, “Kicesie’s Sex Ed” YouTube channel has attracted over 240 million views [31]. In terms of health promotion, the aim would be to achieve viral spread primarily within the target population, as a widely dispersed intervention may be of little value if it does not reach the intended audience.

The challenge for those developing interventions on social networking sites is that no formula exists for achieving viral spread, and we certainly didn’t achieve this with our project (our most popular video had 3118 views). The critical factors believed to be important for viral spread include the structure of the campaign (if it is structured to encourage viral activity, and if it complies to ethical standards), the product being marketed by the campaign (if it is suitable for viral spread) and the message content (if the message is imaginative, contains fun and intrigue, is accessible and is engaging) [32]. Others stress the importance of having individuals with exceptionally large numbers of social connections to share the message [33]. Available empirical data supports that positive content, content inspiring emotion (particularly awe and surprise), content capturing imagination, and the connectivity of the person transmitting the information are important in ensuring viral spread [34-37]. Additionally, viral spread alone may not be enough: while it may increase viewing of one piece of content, this may not translate into sustained interest and engagement. Currently, our best suggestion is to keep trialling different strategies targeted to your audience; hopefully you’ll be lucky and hit the jackpot!

#### Recommendation 7. Define Success and How You Will Measure It

There is little point developing health promotion interventions on social networking sites if it is not possible to measure if they are successful. This brings about two challenges: how to define success and how best to measure it.

As our project was a pilot we had both ‘process’ and ‘impact’ evaluation aims. These evaluation aims included assessing whether we could develop an intervention on social networking sites and attract and engage end users whilst delivering health promotion messages that would have a positive effect on sexual health knowledge and behaviour. As with any approach in its infancy, it is appropriate to focus on process as well as impact evaluation outcomes [38]. 

An appropriate methodology is of critical importance when evaluating interventions on social networking sites. Not only may we wish to evaluate traditional process and impact outcomes for health interventions (eg, reach, dose delivered and received, knowledge and behavioural changes) [38,39], the usability and appeal of the sites is also of key importance. Evaluations of interventions using social networking sites need to appropriately define and measure end user engagement, and develop ways of measuring if and how engagement assists with achieving intervention aims; for example, is a ‘like’ of a page a valid measure of engagement, or is only a comment indicative of true user engagement. Evaluation in this setting is complicated further by the fragmenting of health messages across delivery mechanisms; it can be complex to measure which messages and delivery mechanisms end users were exposed to, and whether this exposure translated into any degree of positive behaviour change.

For our project, we integrated evaluation methods derived from both the health (questionnaires, focus groups, diaries) and information technology (user laboratory testing, expert review) spheres. This is consistent with O’Grady’s proposed ‘dynamic framework’ that suggests incorporating technology (eg, system robustness, reliability, usage statistics) and computer-mediated interaction (eg, usability, accessibility, interactivity) elements within system evaluations [40]. To establish the evidence base for how best to use social networking sites for health promotion interventions, it is critical to move beyond simply collecting end user statistics and integrate evaluation methods from multiple disciplines.

## Conclusion

Although there is much discussion and interest about using social media for health promotion interventions [10,16,17], our experience suggests this is far easier said than done, particularly if the intervention aims to truly use Web 2.0 functions to engage end users. Developing an intervention on social networking sites requires consideration of additional aspects beyond more traditional methods of health promotion. Developers need to consider the online environment and the nature of human interaction online, including Web 2.0 functionality, the characteristics of the target audience and their preferred social networking site(s), and how end users interact and engage in these spaces. Additionally, obtaining ethical, legal and organizational approval, and developing effective evaluation strategies may be challenging. These aspects require additional expertise not typically found in health-focused organizations, and the investment of considerable time and resources.

Social networking sites are now an established part of the online environment; despite being less than ten years old, they are among the most frequently accessed sites globally [13]. While the particular site that is most popular may change over time [12], these sites share common functions that have fundamentally changed how individuals communicate and interact both on- and off-line. Although these sites are primarily used to communicate with social networks, the increasing amount of time individuals spend in these settings [41] suggests that health organizations need to develop effective strategies for reaching individuals in these spaces, whether delivering interventions or using these sites to promote interventions delivered elsewhere.

The FaceSpace Project was our first attempt to develop a health promotion intervention using social networking sites. At the time of project conception there was no information in the published health literature to guide our project development, and undoubtedly we made several mistakes throughout the process. However, our staged implementation approach ensured we could incorporate learnings from the first arm into the second (and now into the extension of the MSM arm), and we were able to develop an appropriate evaluation strategy.

As the popularity of social networking sites continues to increase, we hope that our experience is able to inform the development and evaluation of future health promotion interventions in these spaces. Developing health promotion interventions in this setting, and making mistakes and learning from them is certainly far better than doing nothing at all [30]. With the continuing change in communications media, health organizations must embrace these technologies or risk being left behind.

## Multimedia Appendix 1

Content Monitoring Protocol.

## Abbreviations

MSM: Men who have sex with men
