# Supplementary material for: Developing Health Promotion Interventions on Social Networking Sites: Recommendations from The FaceSpace Project
Source: J Med Internet Res. 2012 Feb 28;14(1):e30. doi: 10.2196/jmir.1875 (PMC3374544; doi:10.2196/jmir.1875)
Supplement: Supplementary file 1 [file jmir_v14i1e30_app1.pdf]

Content Monitoring

In order to ensure that inappropriate content is not being posted on the characters profiles, a two-phase content monitoring strategy will be implemented. This strategy involves an initial exploratory intensive monitoring phase for each implementation arm of the project, whereby all posts will be reviewed within 12 hours of being posted. This monitoring will be performed by the research team at the Burnet Institute (not the actors responsible for the characters).

After the initial two weeks the monitoring approach will be reviewed. If, as expected, few or none of posts have been removed due to inappropriate content, monitoring will become the responsibility of the actor managing the character, who will review all messages posted on their characters profile for inappropriate content each time they log on (every 2-3 days). If any messages are identified as containing potentially inappropriate content, they will be referred to the research team at the Burnet Institute who will review the post within 24 hours and remove it if deemed inappropriate. However, in reality the characters profiles will likely to be monitored on an almost daily basis by the actors managing the characters and the research team monitoring the projects progress.

However, if after the initial two weeks a large number of posts contain inappropriate material, intensive monitoring by the Burnet Institute will be maintained for the duration of the project.

Inappropriate Posts

Posts that may be considered inappropriate and will be removed from the page include:

- Disclosure of information about third parties
- Personal attacks (on the characters or others)
- Sexually explicit material
- Messages posted by someone clearly identified as under the age of 16 years
- Self incriminating information (e.g. admission of drink spiking)

If content is removed, the user posting the content will be sent a

private message informing them of the reason for the removal and a request to refrain from further posting of inappropriate content. If a user repeatedly posts inappropriate content they will be removed from the project (by the character deleting that user as a friend).

Monitoring and Reporting

This two-phase implementation monitoring approach, involving an initial exploratory intensive monitoring phase, appropriately manages the risks of this project and balances the risk-benefit relationship that underpins the ethical review of research, while minimising potential harms to the participant. From the initial two weeks of intensive monitoring, we will be able to more appropriately gauge the probability and severity of users posting any inappropriate material on the characters' profiles, which will then be used to inform further monitoring methods. If throughout this initial trial phase of monitoring, we find that probability and severity of users posting inappropriate material is high, then will continue the intensive monitoring strategy.

It is worth noting that the terms and conditions of the social networking sites stipulate that users must not use the sites to post content which is offensive or harms or threatens any person. The sites retain full discretion to delete or remove (without notice) any content for any reason or no reason, including content that violates the terms and conditions or which might be offensive, illegal, or that might violate the rights, harm, or threaten the safety of users or others. Thus the combined approach of the project monitoring procedures in addition to the existing ongoing monitoring of the social networking sites will ensure that any inappropriate content posted will be removed.

Monitoring and reporting process are as follows:

a) fortnightly reports to the Ethics Committee for the first month
b) monthly reports thereafter until the Committee advises these are no longer needed.

These reports will include information about: Number of fans (total, unsubscribed, removed), demographics of these fans (country, sex, age), wall interactions (posts, comments, likes), views (pages, photos), how many fans have been removed and for what reasons, the time at which unacceptable communications were posted and removed, and the

general character/nature of posts remaining on the characters' pages.
